# Phototoxic damage to cone photoreceptors can be independent of the visual pigment: the porphyrin hypothesis

**DOI:** 10.1038/s41419-020-02918-8

**Published:** 2020-08-29

**Authors:** Mélanie Marie, Valérie Forster, Stéphane Fouquet, Pascal Berto, Coralie Barrau, Camille Ehrismann, José-Alain Sahel, Gilles Tessier, Serge Picaud

**Affiliations:** 1 Sorbonne Université, INSERM, CNRS, Institut de la Vision, 17 rue Moreau, 75012 Paris, France; 2 Université de Paris, Campus Saint Germain, 45 rue des Saints Pères, 75006 Paris, France; 3 Essilor International R&D, 147 Rue de Paris, 94220 Charenton-Le-Pont, France; 4 The University of Pittsburgh School of Medicine, 3550 Terrace Street, Pittsburgh, PA 15213 USA; 5 CHNO des Quinze-Vingts, DHU Sight Restore, INSERM-DGOS CIC 1423, 28 Rue de Charenton, 75012 Paris, France

**Keywords:** Cell death, Cellular imaging, Mechanisms of disease

## Abstract

Lighting is rapidly changing with the introduction of light-emitting diodes (LEDs) in our homes, workplaces, and cities. This evolution of our optical landscape raises major concerns regarding phototoxicity to the retina since light exposure is an identified risk factor for the development of age-related macular degeneration (AMD). In this disease, cone photoreceptors degenerate while the retinal pigment epithelium (RPE) is accumulating lipofuscin containing phototoxic compounds such as A2E. Therefore, it remains unclear if the light-elicited degenerative process is initiated in cones or in the RPE. Using purified cone photoreceptors from pig retina, we here investigated the effect of light on cone survival from 390 to 510 nm in 10 nm steps, plus the 630 nm band. If at a given intensity (0.2 mW/cm²), the most toxic wavelengths are comprised in the visible-to-near-UV range, they shift to blue-violet light (425–445 nm) when exposing cells to a solar source filtered by the eye optics. In contrast to previous rodent studies, this cone photoreceptor phototoxicity is not related to light absorption by the visual pigment. Despite bright flavin autofluorescence of cone inner segment, excitation–emission matrix of this inner segment suggested that cone phototoxicity was instead caused by porphyrin. Toxic light intensities were lower than those previously defined for A2E-loaded RPE cells indicating cones are the first cells at risk for a direct light insult. These results are essential to normative regulations of new lighting but also for the prevention of human retinal pathologies since toxic solar light intensities are encountered even at high latitudes.

## Introduction

Lighting is rapidly changing with the introduction of light-emitting diodes (LEDs) in our homes, workplaces, and cities. These lighting modifications raise major concerns regarding phototoxicity to the retina and other ocular compartments^1,2^. In fact, all external tissues of the body including skin and ocular structures (conjunctiva, cornea, lens, and retina) are daily exposed to natural and artificial lights. Light is a well-known environmental factor to cause diseases of the skin with associated ocular symptoms^3^. Epidemiological studies have also indicated that light, and more precisely blue light, is an identified risk factor for the development of age-related macular degeneration (AMD)^4^. Blue light, whatever its origin (natural sunlight or artificial lightings), carries the highest energy in the visible range and can thus be very deleterious to cells. In addition, many natural molecules like the visual pigment derivative A2E are present in retinal cells and absorb light in the blue range rendering cells highly photosensitive to blue light^5,6^.

To further understand the etiology of AMD, different animal and cell models were used to uncover the mechanisms underlying blue light toxicity. In rodents, rhodopsin and cone pigments have been suggested as the death-mediating chromophores to photoreceptors^7^. More precisely, photoreceptor degeneration was shown to be caused by prolonged or constitutive opsin activation by light^8,9^. Opsin regeneration is required without the need for complete phototransduction cascade^8,10,11^. Consistent with this hypothesis, intermittent blue light exposure induced the death of short wavelength sensitive cones in nonhuman primates whereas green light induced a transient loss of green light sensitive cones^12^. In vitro light damage to cones was only examined using the 661W cell line, which has lost its typical morphology of photoreceptors^13–15^. With these cells, light was found to be more toxic in the presence of all-trans retinal, which is obtained by light conversion of the natural opsin chromophore, the 11-cis retinal^13^.

Because artificial and natural light toxicities were never examined on primary isolated cone photoreceptors, we here investigated the effect of 10 nm wavelength bands onto freshly purified porcine cone photoreceptors^16^. We applied either the same light intensity at all wavelengths to define the most toxic or at intensities of a solar source filtered by the eye optics. Results were interpreted based on autofluorescence examinations of cones and of cone inner segment emission-excitation matrix. Finally, we measured sun light intensities in Paris to compare them with those used in this study.

## Results

### Phototoxicity to isolated cone photoreceptors

**Fig. 1:**
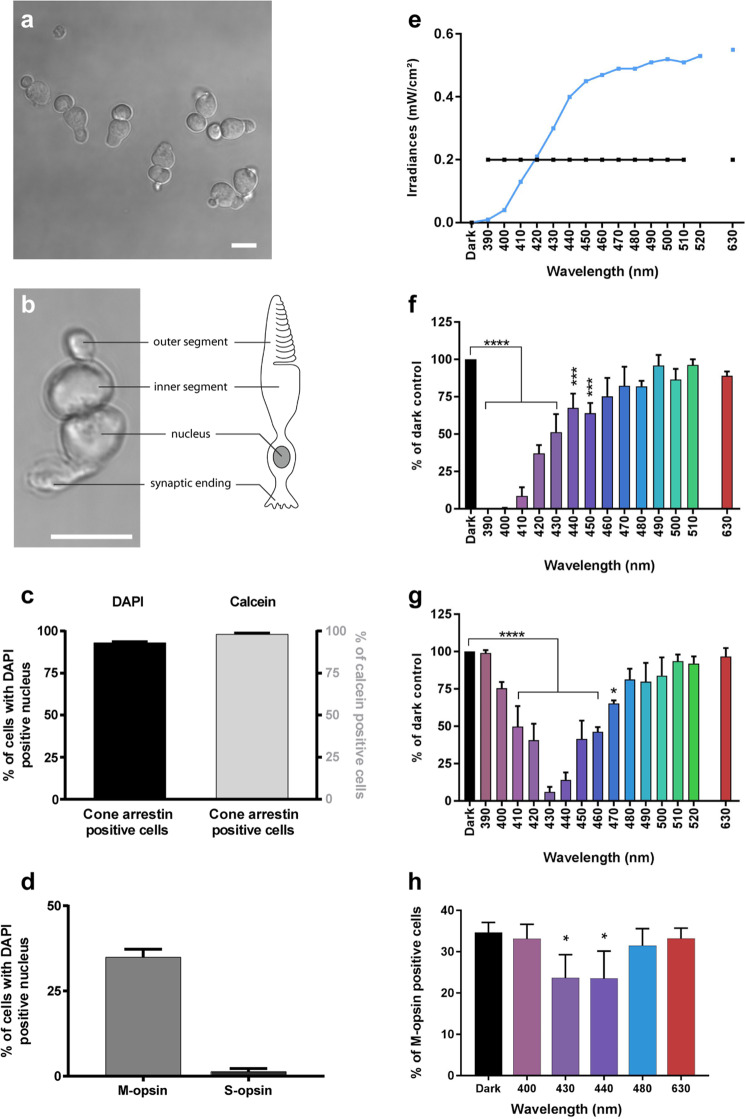
Phototoxicity to isolated primary cone photoreceptors. **a**, **b** Morphology of isolated porcine cone photoreceptors showing outer segment, inner segment, cell body and axon terminal. **c** Characterization of the cell preparation showing that cone arrestin immunopositive photoreceptors represented 93% of purified cells with DAPI-stained nucleus and 98% of the calcein-stained viable cells, *n* = 3. **d** Quantification of green M-opsin and blue S-opsin immune-positive cone photoreceptors in the population among isolated cells, *n* = 3. **e** Irradiance levels in the two sets of experiments, either at identical irradiance (**f**) or normalized to the solar light spectrum reaching the retina (**g**). Spectra of cell viability under the same light irradiance level for all tested 10 nm wavelength bands (**f**) or after normalizing the irradiance levels to the solar spectrum reaching the retina (**g**). Viability was assessed by calcein staining after a 15 h-light exposure to 10 nm-wide bands of light and expressed as percentage of dark control condition. **f** *n* = 3 for 390, 460, 470, 510 nm, *n* = 4 for 410, 420, 450, 490, 500 nm, *n* = 5 for 400, 430, 440, 480, 630. **g** *n* = 5 for 390, 460, 470, 510, 520 nm, *n* = 7 for all other wavelengths. **h** Relative ratio of green M-opsin positive cone photoreceptors in darkness, or after exposure to different wavelengths with irradiance levels normalized to the solar spectrum reaching the retina as in **g**, *n* = 3. Data are expressed as mean ± SEM. Each 10 nm spectral band is designated by its central wavelength. Experiments were performed at least in three independent primary cone photoreceptors preparations. ANOVA followed by Dunnett’s post hoc tests were used to compare variances of all groups. Differences between samples and dark control were considered to be significant when *p* < 0.05 (*), *p* < 0.01 (**), *p* < 0.001 (***) or *p* < 0.0001 (****). Scale bars represent 10 µm.

**Fig. 2:**
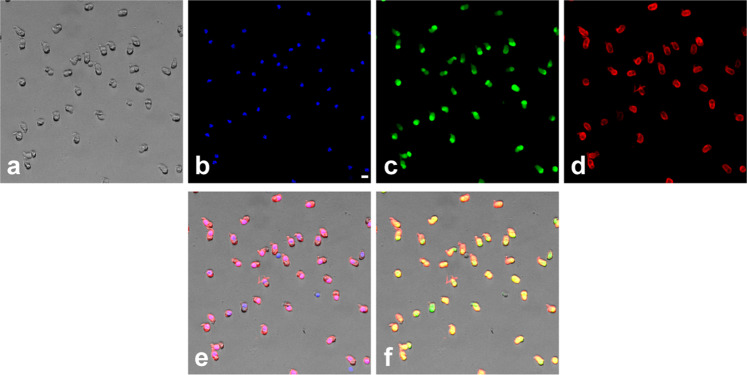
Assessment of cone purity. Cell preparation showing the light transmitted image (**a**), the DAPI nuclear staining (**b**), calcein green staining for viable cells (**c**) and the immunoreactive cone arrestin staining (**d**). The merged images DAPI/cone arrestin (**e**) and calcein/cone arrestin (**f**) indicate that most isolated viable cells are cone photoreceptors as quantified in Fig. 1d. The scale bar represents 10 µm.

**Fig. 3:**
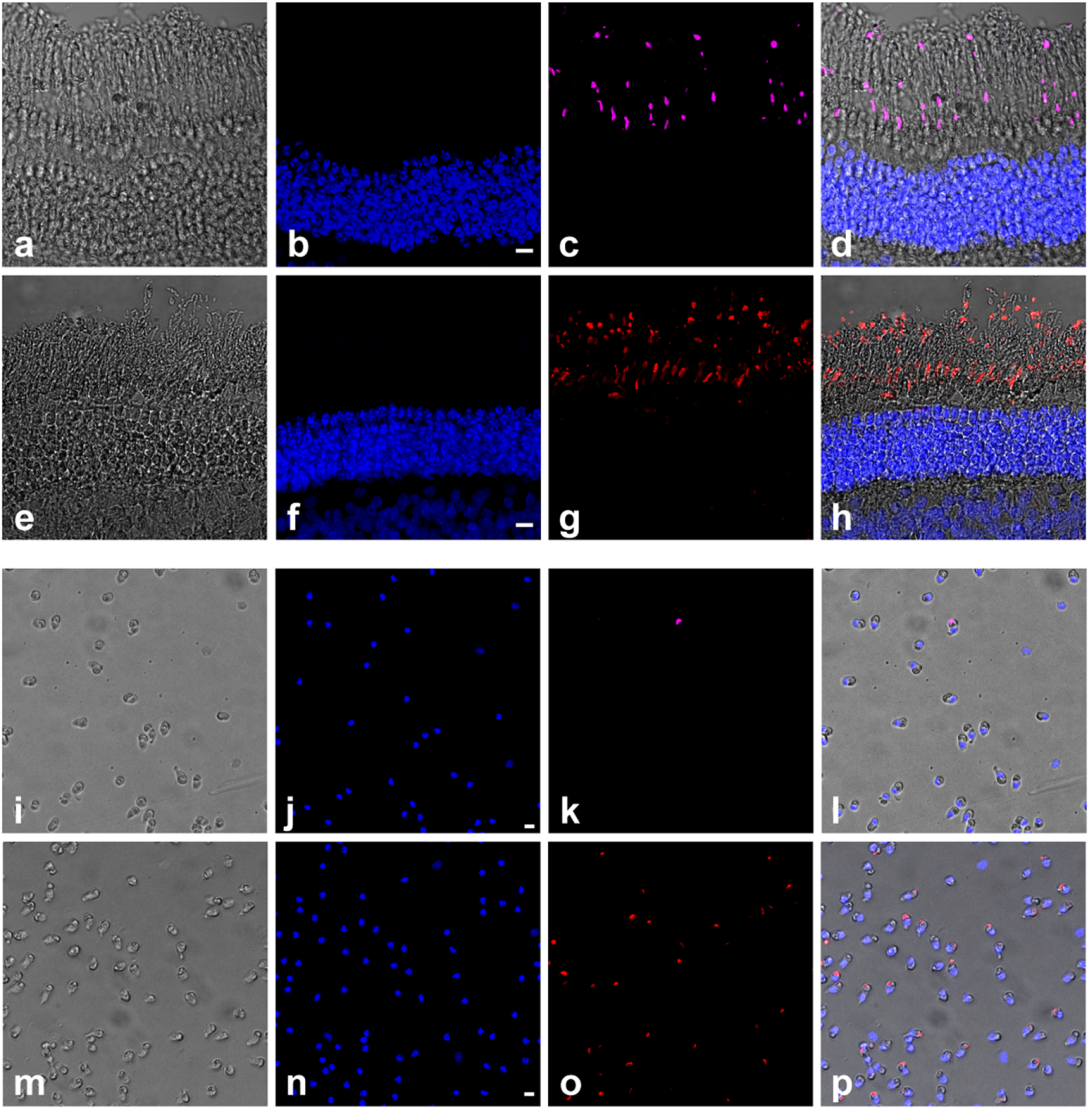
Identification of cone photoreceptors types by immunoreactive opsin staining. Vertical sections (**a**–**h**) and isolated porcine cones (**i**–**p**) showing the blue S-opsin (**c**, **k**) and green M-opsin (**g**, **o**), the DAPI nuclear staining (**b**, **f**, **j**, **n**) the light transmitted images (**a**, **e**, **i**, **m**) and the merged images (**d**, **h**, **l**, **p**). Scale bars represent 10 µm.

To investigate if phototoxicity onto cone photoreceptors could be an early event in ocular diseases and acute light burns, we measured light toxicity on primary isolated cone photoreceptors from pig retinas (Fig. 1a, b) using our dedicated light-emitting device delivering 10 nm-wide bands of light^5^. We first characterized the cell preparation regarding the cell morphology, viability and purity with cone specific markers such as cone arrestin and opsins. Cells exhibited a typical cone photoreceptor morphology with inner and outer segment, cell body and synaptic ending (Fig. 1a, b). The cell preparation was highly enriched with cone photoreceptors since 93% of cells with a DAPI-stained nucleus were immunopositive with a cone arrestin antibody (Figs. 1c, 2a–f). Furthermore, when assessing the cell viability of our preparation with calcein green, we counted that 98% of viable cells were cone-arrestin immunopositive (Figs. 1c, 2a–f). To further characterize the isolated cone photoreceptors, we used cone opsin antibodies against either the blue S-opsin or the green M-opsin. Among isolated cells with a DAPI stained nucleus, 35% of them exhibited an outer segment immunopositive for M-opsin (green cones), only 1.3% of cells exhibited S-opsin immunopositive outer segment (blue cones) (Figs. 1d, 3i–p). Because 98% of purified cells were cone photoreceptors as indicated by their immunolabelling for the cone arrestin protein, the opsin-negative cells, 63% of purified cells, were most likely cone photoreceptors that had lost their outer segment during the dissociation procedure without losing their viability.

We first exposed these purified cones to light at a given irradiance (0.2 mW/cm²) for 15 h for each tested wavelength band (390–510 in 10 nm steps, plus the 630 nm band) to characterize their photosensibility to artificial light (Fig. 1e, f). Blue-violet light (390–450 nm) emerges as highly toxic, with a maximum toxicity between 390 and 410 nm and a toxicity decreasing with increasing radiation wavelength up to 450 nm (Fig. 1f). These data demonstrate the potential toxicity of monochromatic LED light within the near-UV and blue-violet range.

In a second step, we normalized light irradiances to account for eye optic absorption and to evaluate effect of sunlight levels reaching the retina, as previously described^5^. Under these conditions, the most toxic bands were shifted to the 410–460 nm range with a peak of toxicity at 430 and 440 nm (Fig. 1e, g). Indeed, at 430 nm, the survival rate decreased to 6% of the number of cone photoreceptor surviving in darkness. No significant toxicity was observed with green light (Fig. 1g) although the preparation contained 35% M-opsin-positive (green) cones (Fig. 1d). To investigate if the cell toxicity was related to opsin absorption, we quantified the number of surviving cells which were immunopositive for the M-opsin. Surprisingly, the ratio of M-opsin positive cells in surviving cones was decreased with a statistical significance under blue light (430 and 440 nm, Fig. 1h), indicating that the relative loss of these green cones was not correlated to the amplitude of the M-opsin absorption spectrum; the toxicity was greater at 430–440 nm than at 480 nm (Fig. 1h). We attributed this greater blue toxicity onto green-sensitive cones to their cell morphological integrity and the likely light sensitivity of the inner segment (see below). Indeed, the green M-opsin immunolabelling is restricted to the outer segment, therefore it can only be seen in cone photoreceptors with intact outer/inner segments. The absence of greater green light toxicity onto green cones suggests that, in our experimental conditions, light toxicity was independent of the visual pigment.

### Nonvisual pigments and autofluorescence in cone photoreceptors

**Fig. 4:**
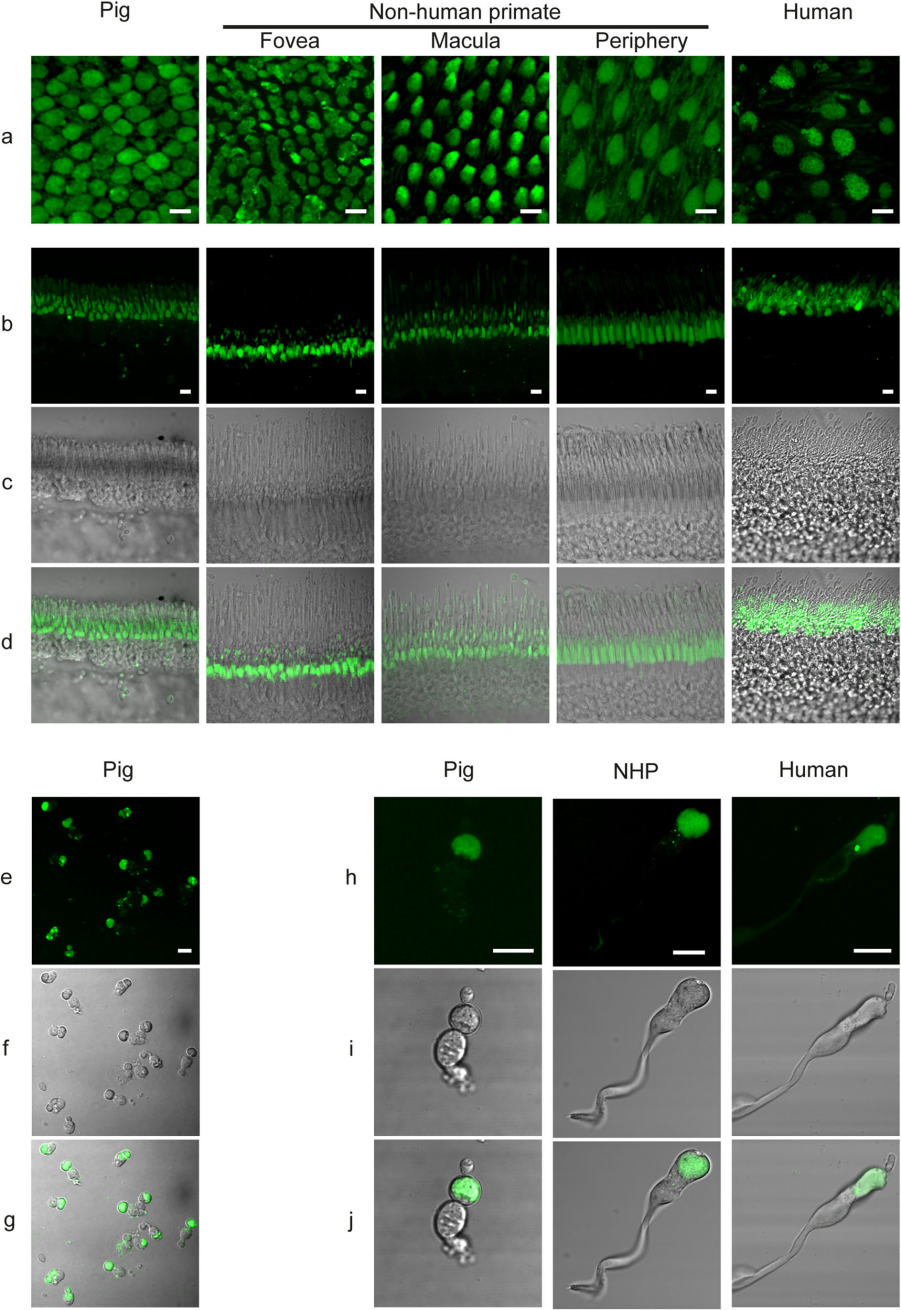
Cone photoreceptor autofluorescence in three mammalian species: pig, nonhuman primate, and human. **a** Live ex vivo flat-mount retinas. **b**–**d** Vertical sections. **e**–**j** Isolated cone photoreceptors. The nonhuman primate tissue (*Macaca fascicularis*) is illustrated at different retinal eccentricities: fovea, peri-macular, periphery. The autofluorescence was measured under a 488 nm laser source. Scale bars represent 10 µm.

**Fig. 5:**
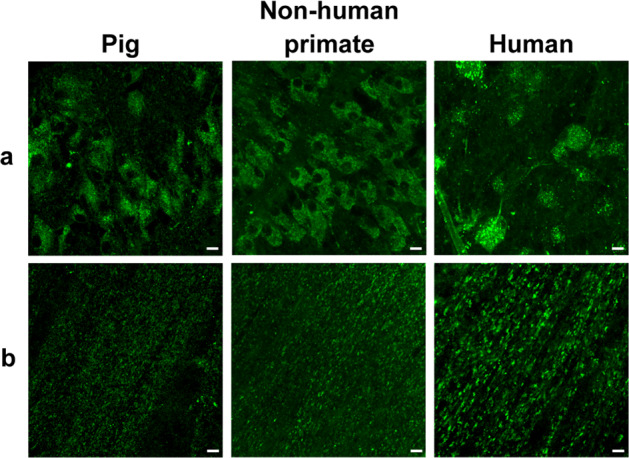
Autofluorescence in ganglion cell and neural fiber layers. Autofluorescence was observed under a 488 nm laser source in the ganglion cell layer (**a**) and in the neural fiber layer (**b**) of three mammalian species: pig, nonhuman primate (*Macaca fascicularis*), and human. Scale bar represents 10 µm.

**Fig. 6:**
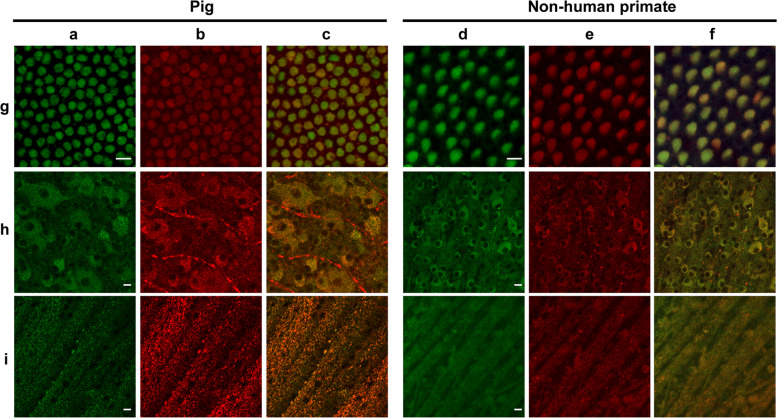
Autofluorescence and mitochondrial colocalization on the flat-mounted retina from pig and nonhuman primate (*Macaca fascicularis*). Autofluorescence (**a**, **d**) and mitochondrial ATP-synthase immunolabelling (**b**, **e**) with the merged images (**c**, **f**) in cone inner segments (**g**), ganglion cell layer (**h**), and optic fiber layer (**i**) showing co-localization except for the unspecific red labeling of blood vessels in the pig ganglion layer (**h**). Scale bars represent 10 µm.

**Fig. 7:**
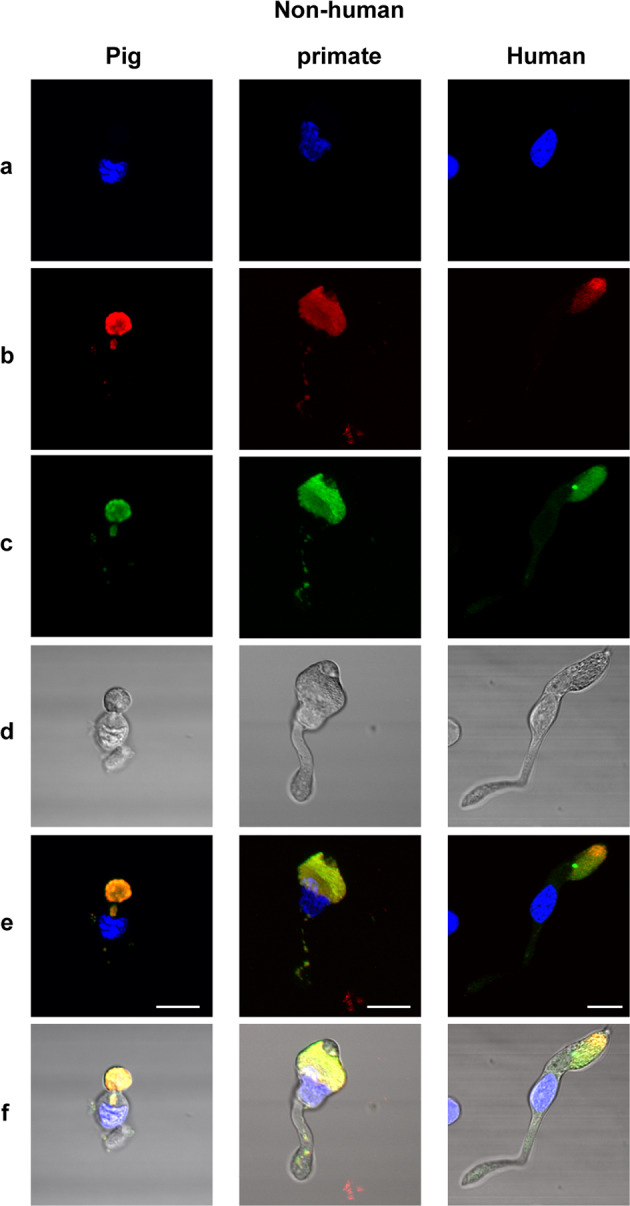
Autofluorescence and mitochondria localization on isolated cone photoreceptors from pig, nonhuman primate (*Macaca fascicularis*), and human retina. DAPI stained nuclei (**a**), mitochondrial ATP-synthase immunolabelling (**b**), autofluorescence (**c**), transmitted light in isolated cone photoreceptors, showing the autofluorescence and ATP-synthase labeling co-localization above the cell body (**e**, **f**). Note that some mitochondria are also seen in the axon of the photoreceptors on the merged images (**e**, **f**). Scale bars represent 10 µm.

To further understand the origin of this phototoxicity, we investigated the presence of other photosensitive pigments in cone photoreceptors. On ex vivo alive retinas, we localized a bright autofluorescence in cone inner segments across species from pigs to nonhuman primates and even humans (Fig. 4a). This bright fluorescence remained on vertical sections that were prepared by vibratome sectioning from the fresh tissue (Fig. 4b, d). Finally, in purified primary cone photoreceptors, the bright fluorescence of their inner segments confirmed the high purity of our preparations from pig, nonhuman primate and even from postmortem human tissue (Fig. 4e, g, h, j). By contrast, rod inner segments generate a weaker autofluorescence, slightly visible on the peripheral flat-mounted retina of the nonhuman primate (Fig. 4b, d). An intense autofluorescence was also localized in cell bodies of retinal ganglion cells (Fig. 5a) and in their axon bundles (Fig. 5b). This autofluorescence in all these structures was attributed to mitochondria because it colocalized to the mitochondrial ATP synthase immunolabelling (Figs. 6, 7).

### Excitation/emission spectra of cone autofluorescence

**Fig. 8:**
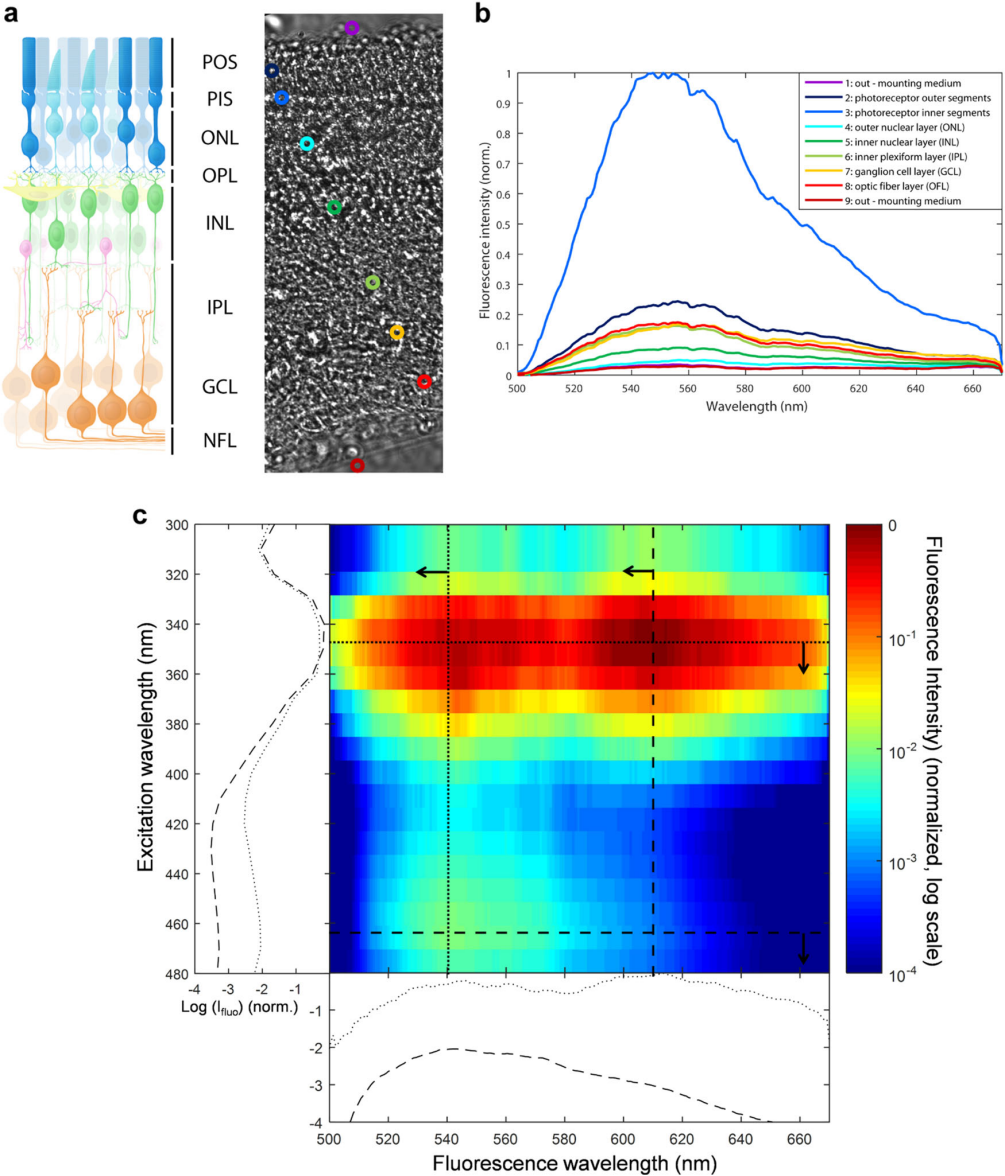
Excitation and emission spectra in the porcine retina. **a** Freshly prepared vertical section of a live pig retina showing the locations used to measure emission spectra presented in **b**. **b** Emission spectra in the different retinal layers showing a peak at 550 nm under a 475 nm laser excitation source. **c** excitation–emission matrix on a cone inner segment exposed to wavelengths ranging from 300 to 490 nm in 15 nm increments. POS: photoreceptors outer segments, PIS: photoreceptor inner segments, ONL: outer nuclear layer, OPL: outer plexiform layer, INL: inner nuclear layer, IPL: inner plexiform layer, GCL: ganglion cell layer, OFL: optic fiber layer.

The autofluorescence in cone photoreceptor inner segment was bright (I_488_) upon 488 nm excitation, weak upon blue excitation at 405 nm (≈33% of I_488_), and below detection thresholds (<1% of I_488_) upon red (594 nm) and far red (644 nm) excitation wavelengths. To investigate the nature of this autofluorescent chromophore, we measured its fluorescence emission spectrum on freshly isolated pig retinas excited by a 475 nm laser source (Fig. 8a, b). Data showed a high fluorescence emission peak centered at 550 nm in the cone inner segment (Fig. 8a, b). This chromophore is unlikely to provoke the light toxicity on cone photoreceptors, as one would have expected to see a peak of toxicity at the absorption peak (488 nm) under the equal illumination irradiances (Fig. 1f). To further define the spectral signatures of chromophores in cones, an excitation–emission matrix was measured in their inner segments (Fig. 8c). Cone inner segments were exposed to wavelengths ranging from 300 to 490 nm in 15 nm increments, and the corresponding emission spectra were recorded. The resulting excitation–emission matrix was normalized to compensate for variations in the intensity of the excitation source (Fig. 8c). Despite a measurement uncertainty below 400 nm due to the lower power of our excitation source, this analysis indicated that the green autofluorescence (540 nm) had two excitation peaks at 350 and 470 nm, which are highly reminiscent of the flavin spectral properties, supporting further a previous proposition for cone in vivo autofluorescence rather than other proposed alternatives^17^. In this analysis, we observed an additional red autofluorescence (610 nm) with an excitation peak at 350 nm (Fig. 8c), which could be attributed to porphyrins^18–20^. This absorption by porphyrins extending in the blue range is consistent with the observed spectrum of phototoxicity on cone photoreceptors (Supplementary information 2). The potential implication of porphyrins in cone phototoxicity is further supported by their known photosensitization^18^.

## Discussion

Phototoxicity of mouse rod photoreceptors was previously attributed to light absorption by rhodopsin^10,11^. We here observed a high toxicity of visible-to-near-UV light to all cone photoreceptors independently of their spectral sensitivity. Blue light was also shown to produce irreversible damage exclusively to short-wavelength sensitive cones in nonhuman primates but green light did not irreversibly damage middle-wavelength cones^12^. This specific blue light damage of short-wavelength cones could be related to their greater vulnerability compared to other cones^21^. The spectrum of cone damage is consistent with the presence of a photosensitive chromophore with a spectral sensitivity in the visible-near-UV range. As cone cell damage was also observed in cones having lost their outer segment, this photosensitive chromophore is likely located outside this specific cell compartment. The measured cone excitation–emission matrix is consistent with high levels of both flavins and porphyrins in cone photoreceptor inner segment. Their location in cone inner segment is in agreement with their high concentrations in mitochondria^22,23^ and with the greater density of these organelles in the cone inner segment^24^. The absence of increased phototoxicity at the blue peak of flavin absorption indicates that the phototoxicity is more likely induced by porphyrins. By contrast, the overlap between published absorption spectra of porphyrins and the light toxicity on cone photoreceptors which we measured at equal irradiance (Supplementary information 2) supports the porphyrin implication. In addition, the phototoxicity of porphyrin derivatives is widely known and taken to advantage in photodynamic therapy to erase cancer cells by light^25–27^.

Blue light has been defined as a risk factor in AMD, which is characterized by the macular loss of cone photoreceptors^4^. Here, normalizing the light irradiance to the solar spectrum reaching the retina, we found out that the most toxic range is located within blue light at 425–445 nm because most of the near-UV light is filtered by the anterior segment of the eye. This toxicity range was evaluated using moderate irradiances (0.39 mW/cm² at 440 nm) and overlaps the toxic light range previously determined on RPE aging models using higher irradiances^5^ (1.09 mW/cm² at 440 nm). A direct cone insult may therefore prevail over the RPE cell damage in the early phases of AMD. Filtering out these blue wavelengths selectively may efficiently protect from AMD development while barely affecting color vision and circadian rhythms.

These findings could be extended to other tissues daily exposed to blue-violet light such as the skin and ocular surface and may explain symptoms in several diseases with an increased sensitivity to photo-oxidative stress^3^. Even if most of the injury is induced by UV-light, our study with a constant spectral irradiance (Fig. 1f) indicates that blue-violet light and thus LED light exposure can contribute to cell damages as reported previously on skin cells^28^. Indeed, visible light penetrates deeper than UV light, and blue-violet light can strongly contribute to oxidative stress in skin cells^28^. In corneal cells at the eye surface, both UV and blue-violet light exposures can contribute to the development of pathologies like the dry-eye syndrome^29,30^.

To investigate the relevance of these results to human pathologies, we compared our toxic light irradiances to daily light levels. The irradiance of 0.39 mW/cm² over a 10 nm light band centered at 440 nm used in vitro corresponds approximately to 0.93 mW/cm² in the same spectral band at the corneal level for a 40-year-old person. Comparatively, on a sunny morning (11:00 a.m.) in Paris during summer (August 29th, 2017), the irradiance level reached 0.46 mW/cm² (Table 1) over the 10 nm-wide band centered at 440 nm when pointing 15° downward a calibrated spectroradiometer, towards low-reflecting surfaces on the fifth floor of a building with an almost clear view, East orientation (Supplementary information 1). Over this very narrow band (440 ± 5 nm), the irradiance measured on a bright morning at the relatively high latitude of Paris (Table 1) is only half the irradiance causing in vitro toxicity in our experiments. Taking into account the effect of other toxic wavelength bands (410–460 nm), lower latitudes, or higher albedos, a pedestrian can encounter toxic blue light levels on a sunny summer day. Fortunately, the 15-h exposition inflicted on isolated cone photoreceptors is unlikely, but cumulative effects over a week could become toxic since mitochondrial renewal is not finalized in a day: complete mitochondrial fusion–fission cycles were reported to take 16 days in the adult mouse^31^. These data strongly indicate that cone phototoxicity can occur even at moderate latitudes, or under artificial lighting.

**Table 1:** Comparison of light irradiances used for in vitro experiments and measured outdoor in Paris.

Wavelength (nm)	Cone irradiances in vitro (mW/cm²)	Corresponding cone irradiances at the corneal level in a 40 year-old eye (mW/cm²)	Irradiances at the corneal level measured in Paris August 29th, 2017, 11:00 am, East orientation, 5th floor of a building with an almost-clear view, sensor downard −15° towards a low reflecting ground (mW/cm²)
400	0.04	2.00	0.31
410	0.13	1.86	0.38
420	0.22	1.22	0.39
430	0.31	0.97	0.39
440	0.39	0.93	0.46
450	0.48	1,00	0.53
460	0.44	0.83	0.56
470	0.45	0.80	0.58
480	0.56	0.95	0.59
490	0.54	0.87	0.57
500	0.51	0.78	0.61
510	0.50	0.75	0.59
520	0.69	1.00	0.60
630	0.56	0.69	0.60

Comparison between irradiance levels integrated over 10 nm-wide light bands (designated by their central wavelengths) on isolated cones, their correspondence at the corneal level for the different wavelengths and measured outdoor irradiances in Paris on a sunny morning (11:00 am) on August 29th, 2017, East orientation, 5th floor of a building, light sensor oriented at −15° downward.

This cell toxicity mechanism can easily explain retinal damage observed when staring at the sun during solar eclipses^32^ or following indirect ground reflection in more exposed countries^33,34^. This photosensitization could also accelerate the degenerative process in the context of retinal diseases like retinitis pigmentosa showing greater sensitivity of cones to oxidative stress^24^. Because the described phototoxicity of cone photoreceptors can affect all the population including children, it is further reinforcing the concerns raised by the use of high-intensity LEDs with high blue contents^1,2^.

## Materials and methods

### Mammalian retinas

Porcine eyes were bought at a local slaughterhouse in agreement with the local regulatory department and the slaughterhouse veterinarians (agreement FR75105131). This procedure adheres to the European initiative for restricting animal experimentation because not a single animal was euthanized for our experimentation. Eyes were taken from animals daily sacrificed for food production.

Nonhuman primate retinas were prepared using eyes received from adult macaques (*Macaca fascicularis*) that were terminally anesthetized for unrelated studies. All animal experiments and procedures were ethically approved by the French “Ministère de l’Education, de l’Enseignement Supérieur et de la Recherche” and were carried out according to institutional guidelines in adherence with the National Institutes of Health guide for the care and use of laboratory animals as well as the Directive 2010/63/EU of the European Parliament.

Postmortem human ocular globes from donors were acquired from the School of Surgery (Ecole de Chirurgie, Assistance Publique Hôpitaux de Paris, Paris, France, CODECOH DC-2015–2400). The protocol was approved by the IRBs of the School of Surgery and the Quinze-Vingts National Ophtalmology Hospital (Paris, France). All experiments on postmortem human retinal explants were performed according to the local regulations, as well as the guidelines of the Declaration of Helsinki.

### Tissue preparation

Eyes were cleaned up from muscles and incubated during 2 min in Pursept-AXpress (Thermo Fisher Scientific, Waltham, MA, USA) for disinfection. The anterior segment was cut along the limbus to remove the cornea, lens and vitreous. The retina was carefully removed from the eyecup and placed into CO_2_ independent medium (Life Technologies, Carlsbad, CA, USA). A 1 cm² piece was cut and either immediately flat-mounted in Permafluor medium (Thermo Fisher Scientific) or included in 2–4% low melting agarose dissolved in a pre-warmed (+37 °C) custom modified Neurobasal A medium without any photosensitizer such as phenol red, riboflavin, folic acid and aromatic amino acids (modified NBA, Life Technologies) for retinal sectioning. Transversal slices of 100 µm thickness were cut using a vibrating microtome (Leica, Wetzlar, Germany) in modified NBA medium and then mounted in Permafluor medium (Thermo Fisher Scientific). Images were acquired without further delays.

### Cell isolation

Cone photoreceptors were purified by lectin-panning selection as previously described^16^ and seeded in black clear bottom 96-well plates in a custom modified Neurobasal A medium without any photosensitizer such as phenol red, riboflavin, folic acid and aromatic amino acids (modified NBA, Life Technologies) for light exposure. For live imaging, cells were isolated on specific 35 mm Petri dish with high optical quality bottom for microscopy (IBIDI, Munich, Germany).

### Light conditions

Cells were exposed to 10 nm-wide light bands produced by a custom-made LED-based fibered illumination device for 15 h as previously described by^5^. Each light band is designated by its central wavelength. Central wavelengths of the narrow light bands were equally distributed from 390 to 520 nm in 10 nm increments (14 narrow bands available). A 15th band with a central wavelength set at 630 nm was added. To model physiological light conditions reaching the retina, irradiances for each band of light were calibrated according to a normalized daylight spectrum obtained by applying the natural ocular media filtering of a 40-year-old eye (CIE 203:2012) onto the referenced solar spectrum (ASTM, G173-03, International standard ISO 9845-1, 1992^5,35^). Accordingly, the 10-nm irradiances varied from 0.01 mW/cm² at 390 nm to 0.56 mW/cm² at 630 nm, with 0.39 mW/cm² at 440 nm (Supplementary Fig. S6). Irradiances and spectra were measured using the calibrated spectroradiometer JAZ (Ocean Optics, Dunedin, USA). After a 4-h rest period following seeding in a 96-well plate, cone photoreceptors were either exposed to the physiological solar spectrum or to 0.2 mW/cm² for each band of light. After light exposure, cells were maintained in darkness for 24 h before viability measurement or fixation for immunocytochemistry. Irradiance measurements in real life at the corneal level were assessed using the spectroradiometer JAZ oriented downward with a −15° toward a low-reflecting ground (to mimic the head inclination of a person walking) on the fifth floor of a building (GPS coordinates: 48.84983 and 2.372486) with an almost clear view, East orientation, in a sunny morning (11:00 a.m.) in Paris during the summer (August 29th, 2017). Spectral irradiances (mW/cm²) were measured with a 1 nm step and then integrated over 10 nm bandwidths to compare to the in vitro irradiances.

### Viability measurement

To assess viability, calcein (Life Technologies) was added to the cell culture medium 24 h after the end of light exposure and incubated during 1 h. The number of positive viable cells was then counted with an automated microscope equipped with a ×10 objective (Arrayscan, Thermo Scientific, Rockford, IL, USA). Figure 1f*n* = 3 for 390, 460, 470, 510 nm, *n* = 4 for 410, 420, 450, 490, 500 nm, *n* = 5 for 400, 430, 440, 480, 630. Figure 1g*n* = 5 for 390, 460, 470, 510, 520 nm, *n* = 7 for all other wavelengths. Experiments were excluded when surviving cells in the dark control condition were 25% below the number of viable seeded cells, indicating a poor quality of the provided retina. Mean of the surviving cells observed in dark control was 50% of seeded cells.

### Immunocytochemistry

Cone photoreceptors were either fixed in the 96-well plates or Petri dish with 4% paraformaldehyde after cell isolation during 15 min at room temperature (PFA, Sigma-Aldrich, St Louis, MO, USA) or 24 h after the end of light exposure during 1 h at room temperature. Cells were then washed three times with phosphate buffer saline (PBS) before being permeabilized with PBS 0.1% Triton X-100 (Sigma) for 3 min at room temperature and washed again three times in PBS. To prevent nonspecific binding cells were incubated during 2 h at room temperature in a blocking buffer solution containing 1% BSA (Bovine Serum Albumin, Sigma-Aldrich), 0.05% Tween 20 (Sigma-Aldrich) in PBS. Cells were immuno-stained overnight at +4 °C with a rabbit polyclonal antibody raised against S-opsin (Merck-Millipore, Billerica, MA, USA), a rabbit polyclonal antibody raised against M-opsin (Merck-Millipore), with a rabbit polyclonal antibody directed against the human cone arrestin (LUMIf-hCAR/human cone arrestin (ARR4), Cheryl Mae Craft, University of Southern California Roski Eye Institute, Los Angeles, CA^36,37^) or with a mouse antibody raised against ATP synthase subunit b (Life Technologies). After primary immunostaining, cells were washed several times with PBS and incubated for 2 h at room temperature with either anti-rabbit Alexa 594 or anti-mouse Alexa 594 secondary antibodies (Life Technologies). All antibodies were diluted in the blocking buffer solution. Nuclei were counterstained with 4′,6-Diamidine-2′-phenylindole dihydrochloride (DAPI, Sigma-Aldrich).

### Immunohistochemistry

For opsin labeling, porcine eyes without anterior segment were fixed with 4% paraformaldehyde overnight at +4 °C. Eyes were cryoprotected in successive solutions of PBS containing 10, 20, and 30% sucrose at +4 °C before being embedded in NEG50 (Microm, Francheville, France). Retinal cryosections (12 µm) were cut using a cryostat (Microm). For retinal flatmount immuno-labeling, freshly isolated retina were fixed with 4% paraformaldehyde during 1 h at room temperature. Retinal piece or sections were incubated 2 h at room temperature in blocking buffer solution containing PBS, 1% Triton X-100, 5% BSA, 0.5% Tween 20. Retinal tissues or cryosections were immune-stained overnight at +4 °C with a rabbit polyclonal antibody raised against S-opsin (AB5407, Merck-Millipore, Billerica, MA, USA), a rabbit polyclonal antibody raised against M-opsin (AB5405, Merck-Millipore), or with a mouse antibody raised against ATP synthase subunit b (A-21351, Life technologies). All antibodies were diluted in the blocking buffer solution. After primary immunostaining, retinal tissues and cryosections were washed several times with PBS and incubated for 2 h at room temperature with either anti-rabbit Alexa 594 or anti-mouse Alexa 594 secondary antibodies (Life Technologies). Nuclei were counterstained with 4′,6-Diamidine-2′-phenylindole dihydrochloride (DAPI, Sigma-Aldrich). Retinal tissues and cryosections were then mounted in Permafluor medium (Thermo Fisher Scientific).

### Opsin quantification

After immunocytochemistry, M-opsin and S-opsin positive cells were quantified among purified cone photoreceptors in 96 well-plates using an automated microscope equipped with a ×10 objective (Arrayscan, Thermo Scientific).

### Confocal imaging

Imaging was performed either on freshly isolated cells or tissues without any fixation step or on immunolabelled samples using an Olympus FV1200 (or FV1000) laser-scanning confocal microscope. DAPI counterstaining, autofluorescence, and AlexaFluor-594 were excited using 405 nm laser diode, 488 nm argon ion laser, and 559 nm laser diode lines, respectively. Selections of excitation wavelengths and emission wavelengths were controlled by appropriate filters: dichroic miror (405/488/559/635), emission beam splitters SDM490 (or spectral detection), SDM560 (or spectral detection), SDM640, and barrier filters BA430–470, BA505–540, BA575–675, and BA655–755, respectively. The objectives used were water dipping objective LUMPLFLN 40X NA 0.8-WD 3.3, oil immersion PLAPON 60X SC NA1.4-WD 0.12, UPLSAPO 100X NA 1.4 WD 0.13. Control of the microscope and image acquisition were conducted using Olympus Fluoview software version 4.2. Image acquisition was conducted at a resolution of 1024 × 1024 pixels, with a scan rate of 10 μs pixel^−1^. Images were acquired sequentially, line by line, in order to reduce excitation and emission crosstalk, and step size was defined according to the Nyquist–Shannon sampling theorem. Exposure settings that minimized saturated pixels in the final images were used. Twelve bit images were then processed with ImageJ or FIJI, Z-sections were projected on a single plane using maximum intensity under Z-project function and finally converted to 24 bits RGB color images. Figures were then assembled by using Adobe Photoshop CC and Adobe Illustrator software (Adobe, San Jose, CA, USA).

### Fluorescence excitation and emission spectra

To define the spectral signature of chromophore in cone photoreceptors, freshly isolated retinas were transversally sectioned using a vibrating microtome as described above, a section was place between slide and coverslip in Permafluor mounting medium to expose cone inner segments to wavelengths ranging from 300 to 490 nm in 15 nm increments. The corresponding emission spectra were recorded using focused excitation, a high-λ-pass filter to remove the excitation, and confocal detection with an Andor Shamrock SR303i spectrometer and Andor Ixon 3 EM-CCD (Andor Technology, Belfast, Northern Ireland). The resulting excitation–emission matrix was normalized by the excitation intensity to compensate its spectral variations, although this increases noises below 400 nm, where the available excitation intensity is weaker.

### Statistics

Experiments were repeated at least three times. Results for cell viability and cell type quantifications after light exposure were therefore the averaged values of at least three independent experiments (represented biological replicates). For cell viability measurement, each experiment was the mean of at least three technical replicates. The data were normalized to dark control and represented as mean ± SEM. Statistical analyzes were performed using GraphPad Prism 7.03 Software (GraphPad Prism, La Jolla, CA, USA). One-way ANOVA followed by Dunnett’s post hoc tests were used to compare variances of all groups. Differences between samples and dark control were considered to be significant when *p* < 0.05 (*), *p* < 0.01 (**), *p* < 0.001 (***) or *p* < 0.0001 (****).

## Supplementary information

Supplementary information 1

Supplementary information 2

Supplementary information
