# Supplementary figures and images for: Phototoxic damage to cone photoreceptors can be independent of the visual pigment: the porphyrin hypothesis
Source: Cell Death Dis. 2020 Aug 29;11(8):711. doi: 10.1038/s41419-020-02918-8 (PMC7456424; doi:10.1038/s41419-020-02918-8)

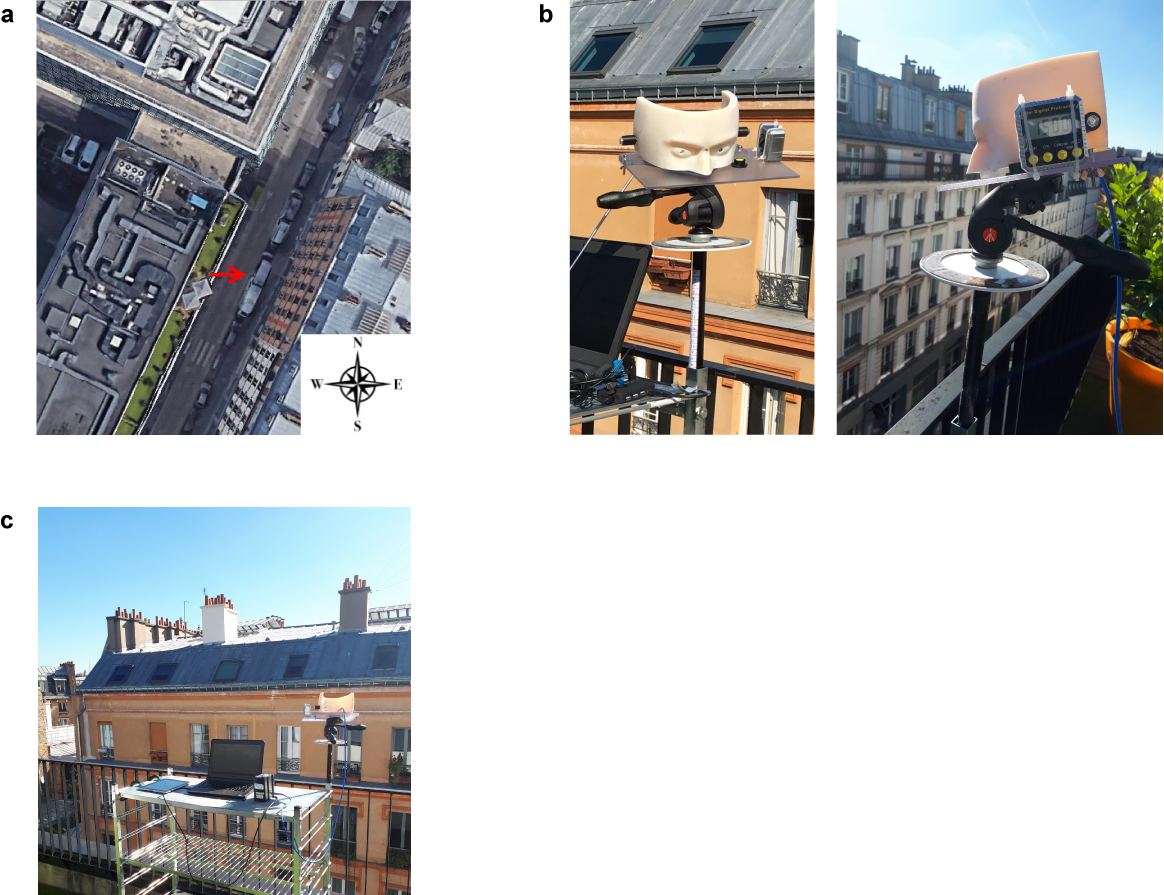

Supplement: Supplementary file 1 — Supplementary information 1 [file 41419_2020_2918_MOESM1_ESM.tif]

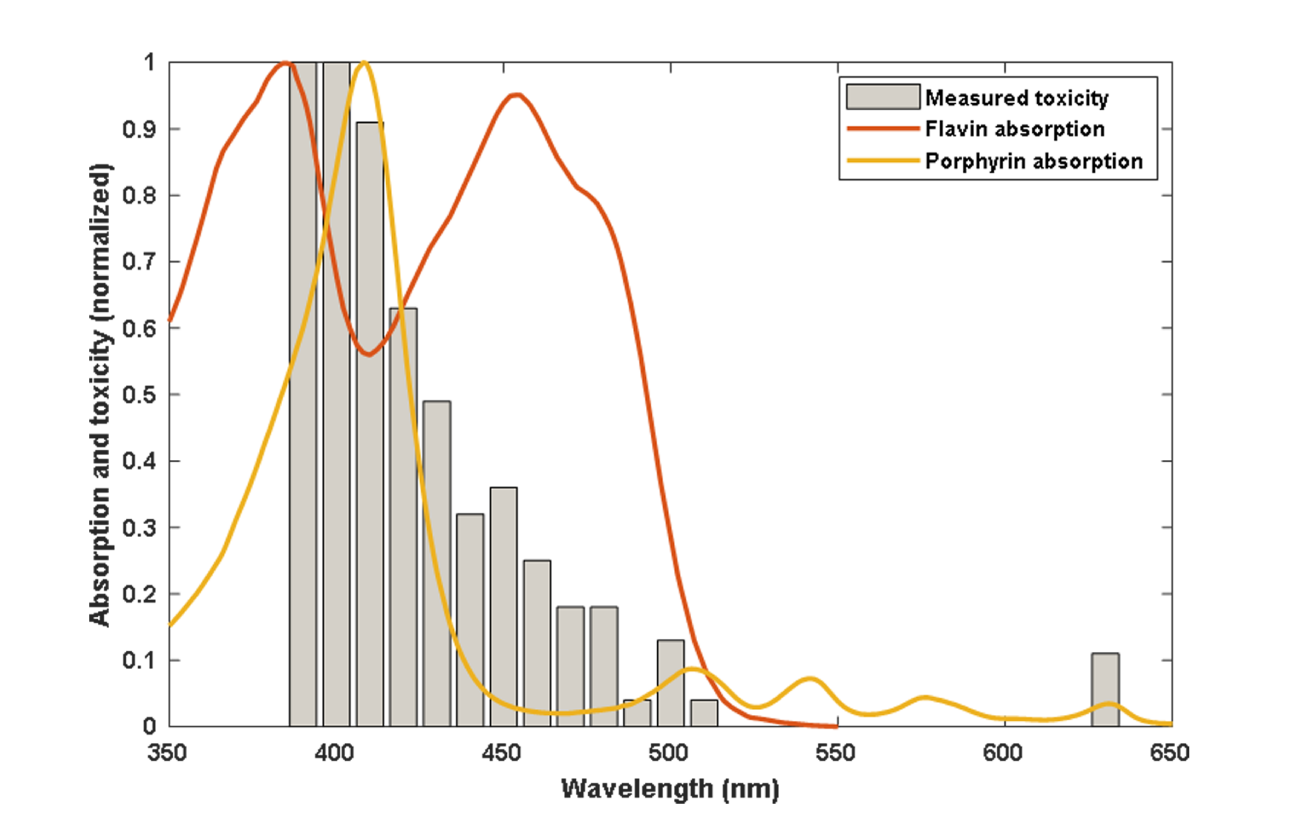

Supplement: Supplementary file 2 — Supplementary information 2 [file 41419_2020_2918_MOESM2_ESM.tif]
