# Supplementary material for: Phototoxic damage to cone photoreceptors can be independent of the visual pigment: the porphyrin hypothesis
Source: Cell Death Dis. 2020 Aug 29;11(8):711. doi: 10.1038/s41419-020-02918-8 (PMC7456424; doi:10.1038/s41419-020-02918-8)
Supplement: Supplementary file 3 — Supplementary information [file 41419_2020_2918_MOESM3_ESM.docx]

**Supplementary information**

**Phototoxic damage to cone photoreceptors can be independent of the visual pigment: the porphyrin hypothesis**

Mélanie MARIE^1,*^, Valérie FORSTER^1,*^, Stéphane FOUQUET^1^, Pascal BERTO^1,2^, Coralie BARRAU^3^, Camille EHRISMANN^3^, José-Alain SAHEL^1,4,5^, Gilles TESSIER^1^, Serge PICAUD^1^

^*^These authors contribute equally to this work

**Supplementary information 1: Optical set-up for real life irradiance measurements, in Paris.** Localization of the optical set-up (GPS coordinates: 48.84983 | 2.372486), red arrow represents gaze direction (a). A 3D-printed head model was set at 1.6 meters from the floor to measure light irradiances. The head model was oriented downward with a -15° with the light sensor (cosine corrector) of the calibrated spectroradiometer in the eye (b). Spectral irradiances (mW/cm²) were recorded and integrated over each 10-nm wide light band (b and c). Complete optical bench showing the sight direction of the head model, East orientation, in a summer and sunny morning in Paris, watching the building on the opposite side of the street (c).

**Supplementary information 2:** Comparison of measured cone toxicity to the absorption spectra of flavins and porphyrins. Grey bars: phototoxicity measured on primary cone photoreceptors submitted to illumination at various wavelengths with the same irradiance level (data from Fig. 1f applying the equation “toxicity = 1 – viability”). Red line: normalized flavin absorption, taken from Kennis *et al.* 2013. Yellow line: normalized porphyrin absorption, taken from Falk *et al.* 1964. Although no measurements could be made below 390 nm and in the 520-620 nm region, the clear overlap between toxicity and porphyrin absorption definitely points towards a toxicity mechanism involving porphyrins.

References: Falk, J. E. (1964) Porphyrins and metalloporphyrins. Their General, Physical and Coordination Chemistry and Laboratory Methods, p. 232, Elsevier; Kennis, JTM and Mathes, T (2013) Molecular eyes: proteins that transform light into biological information, Interface FocusVolume 3, Issue 5
